# Early atrial remodeling predicts the risk of cardiovascular events in patients with metabolic syndrome: a retrospective cohort study

**DOI:** 10.3389/fcvm.2023.1162886

**Published:** 2023-05-03

**Authors:** Rohbaiz Wali, Xinying Wang, Chenglin Li, Heng Yang, Fei Liu, Salah D. Sama, Lan Bai, Sharen Lee, Tesfaldet H. Hidru, Xiaolei Yang, Yunlong Xia

**Affiliations:** ^1^ Department of Cardiology, First Affiliated Hospital of Dalian Medical University, Dalian, China; ^2^ Yidu Cloud Technology, Ltd., Beijing, China; ^3^ Faculty of Medicine, The Chinese University of Hong Kong, Hong Kong, Hong Kong SAR, China

**Keywords:** atrial myopathy, metabolic syndrome, left ventricular hypertrophy, cardiovascular diseases, atrial remodeling

## Abstract

**Background:**

This study aims to assess the prevalence of atrial cardiomyopathy (ACM) in patients with new-onset metabolic syndrome (MetS) and investigate whether ACM could be a predictor of hospital admission for cardiovascular (CV) events.

**Methods:**

Patients with MetS who were free of clinically proven atrial fibrillation and other CV diseases (CVDs) at baseline were included in the present study. The prevalence of ACM was compared between MetS patients with and without left ventricular hypertrophy (LVH). The time to first hospital admission for a CV event between subgroups was assessed using the Cox proportional hazard model.

**Results:**

A total of 15,528 MetS patients were included in the final analysis. Overall, LVH patients accounted for 25.6% of all newly diagnosed MetS patients. ACM occurred in 52.9% of the cohort and involved 74.8% of LVH patients. Interestingly, a significant percentage of ACM patients (45.4%) experienced MetS without LVH. After 33.2 ± 20.6 months of follow-up, 7,468 (48.1%) patients had a history of readmission due to CV events. Multivariable Cox regression analysis revealed that ACM was associated with an increased risk of admission for CVDs in the MetS patients with LVH [hazard ratio (HR), 1.29; 95% confidence interval (CI), 1.142–1.458; *P* < 0.001]. Likewise, ACM was found to be independently associated with hospital readmission due to CVD-related events in MetS patients without LVH (HR, 1.175; 95% CI, 1.105–1.250; *P* < 0.001).

**Conclusion:**

ACM is a marker of early myocardial remodeling and predicts hospitalization for CV events in patients with MetS.

## Introduction

The burden of cardiovascular disease (CVD) is expected to more than double in the next three decades because of the increased average global life expectancy (1). Consequently, the rate of hospitalization due to CVD in healthcare facilities is equally expected to increase. This demands intensive efforts from the scientific community to identify predictors or indicators of the risk of hospitalization due to CVD.

Metabolic syndrome (MetS) is a cluster of metabolic disorders including glucose intolerance, low levels of high-density lipoprotein cholesterol (HDL-C), high levels of triglycerides (TG), obesity, and hypertension (HTN) (2, 3). MetS, frequently combined with other cardiovascular (CV) risk factors, increases CV morbidity and mortality (4). There is emerging evidence that MetS is associated with left ventricular hypertrophy (LVH), a hallmark of preclinical CV diseases (CVDs) (5). However, LVH is not a sensitive marker for myocardial damage.

Atrial cardiomyopathy (ACM), the structural and pathophysiologic changes in the atria, can lead to sustained cardiac arrhythmia. Such dysrhythmia is denoted as atrial fibrillation (AF) (6). The underlying mechanisms involving ACM are atrial dilation (7), fibrosis (8), endothelial cell dysfunction (9), and impaired myocyte function (10). Various electrocardiographic (ECG), echocardiographic, and serum markers have been found to be associated with ACM, such as increased P-wave terminal force in V1 (PTFV1) (11), paroxysmal supraventricular tachycardia (12), premature atrial contraction (13), increased PR interval (14), increased left atrial (LA) size (15) or volume (16), and elevated N-terminal pro-B-type natriuretic peptide (16).

Recently, there has been an increased interest in the diagnostic value of LA remodeling in myocardial injury. This may be attributed to the fact that LA enlargement (LAE) occurs earlier than LVH and is regarded as an independent risk factor for CV events (17). Understanding the prevalence and impact of ACM in MetS may shed light on the risk of hospitalization due to CVDs. Thus, here, we aimed to determine the prevalence of ACM and its effect on the risk of hospitalization for CV events in patients with MetS.

## Methods

### Study design and participants

**Figure 1:**
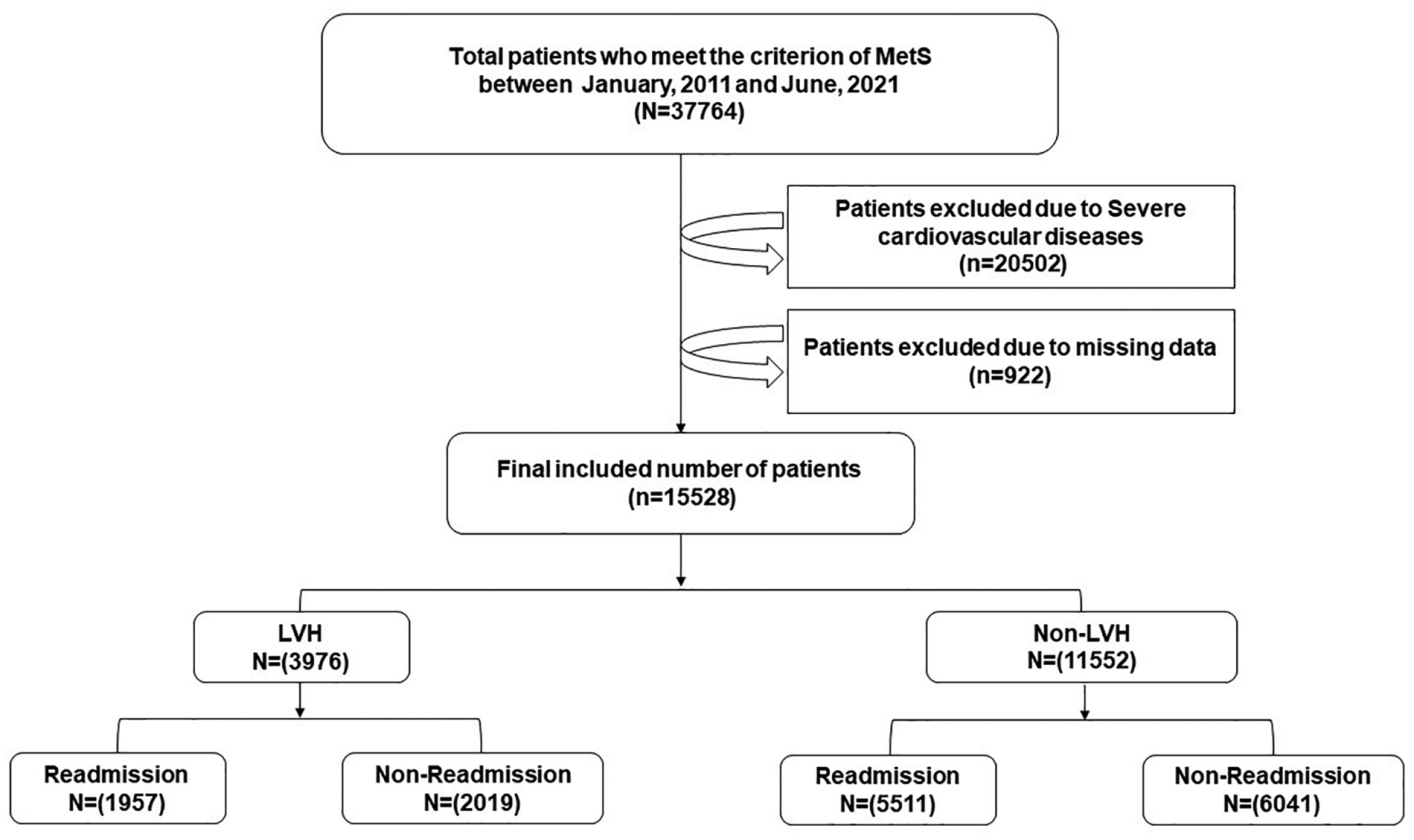
A brief overview of the selection of study participants. MetS, metabolism syndrome; LVH, left ventricular hypertrophy.

This retrospective cohort study was conducted on the basis of data obtained from the Electronic Medical Record Research Database (EMRRD) of the First Affiliated Hospital of Dalian Medical University (FAHDM). The EMRRD was developed to establish a computerized clinical database, and the clinical records are continuously updated (18). A total of 37,764 patients who experienced MetS and were hospitalized at the FAHDM between 1 January 2011 and 31 June 2021 were initially recruited. Patients with a history of AF, secondary HTN, coronary heart disease (including a history of angina pectoris, myocardial infarction, coronary revascularization, or more than 50% narrowing of one of the epicardial coronary arteries on coronary computed angiography), heart failure, cardiac valvular stenosis, moderate or severe valvular regurgitation, cardiomyopathy, severe hepatic and renal dysfunction, and malignant tumor, and/or whose data were missing or contained errors were excluded from the study. After excluding patients who fulfilled the exclusion criteria, a total of 15,528 patients were included in the analysis (Figure 1).

### Evaluation of the metabolic syndrome

Using the National Cholesterol Education Program Third Adult Treatment Panel (NCEP-ATP III) guidelines, MetS was defined on the basis of the presence of three or more of the following: increased waist circumference [≥90 cm in men or ≥85 cm in women], elevated TG [≥150 mg/dL (1.7 mmol/L) or drug treatment for elevated TG], low HDL-C [<40 mg/dL (1 mmol/L) in men and <50 mg/dL (1.3 mmol/L) in women or medical treatment for low HDL-C], elevated blood pressure [systolic blood pressure (SBP) ≥130 mmHg or diastolic blood pressure (DBP) ≥85 mmHg or current use of antihypertensive medication], and impaired fasting glucose [fasting plasma glucose ≥100 mg/dL (5.6 mmol/L) or current use of antidiabetic medication] (19).

### Echocardiographic assessment

All subjects underwent transthoracic echocardiography at rest in the left lateral decubitus position using the Vivid 7 ultrasound system (GE Vingmed Ultrasound, Horten, Norway). Standard two-dimensional echocardiography with Doppler examination was performed, and measurements were obtained according to the guidelines of the American Society of Echocardiography (20). The left atrium diameter was obtained as previously reported (21), and LAE was defined on the basis of a posteroanterior dimension >35 mm. The left ventricular mass was measured by echocardiography, and the left ventricular mass index was calculated (22). LVH was defined on the basis of the following parameters: LVMI >115 g/m^2^ for men and >95 g/m^2^ for women (23). Experienced ultrasound experts who were blinded to the clinical data reviewed the echocardiography results.

### Covariates, follow-up, and clinical outcomes

PTFV1 was calculated for all patients using standard digital 12-lead ECGs on admission and was obtained using digital calipers, measuring the absolute value of the depth (μV) times the duration (ms) of the downward deflection (terminal portion) of the P-wave in lead V_1_. ACM was defined as PTFV1 >4,000 μV·ms or severe LAE (24, 25). A patient was considered to have HTN if a systolic SBP ≥140 mmHg, a mean DBP ≥90 mmHg, and/or current use of an antihypertensive drug were shown in their medical history (26). According to the 2016 Chinese guidelines for the management of dyslipidemia in adults, dyslipidemia was defined on the basis of the following parameters: total cholesterol ≥6.22 mmol/L, low-density lipoprotein cholesterol (LDL-C) ≥4.14 mmol/L, HDL-C <1.04 mmol/L, TG ≥2.26 mmol/L, or an indication of the use of lipid-lowering drugs (27). Diabetes mellitus (DM) was defined in terms of fasting plasma glucose ≥126 mg/dL or treatment with insulin or oral hypoglycemic medication (28). Smoking was defined in terms of current smoking status or a lifetime consumption of >100 cigarettes.

The study endpoint was hospitalization for any CV event (acute myocardial infarction, congestive heart failure, ischemic stroke, or atrial fibrillation) during the follow-up period. Data were analyzed according to the number of hospitalizations for each CV event. The follow-up period was the time starting from the index date to the occurrence of hospitalization for a CV event or to the end of this period (31 October 2021), whichever came first.

### Statistical analysis

Continuous variables with a normal distribution pattern are expressed as means ± standard deviations, whereas variables with a non-normal distribution pattern are presented as medians with 25th and 75th percentiles. For categorical variables, the chi-square test (*χ*^2^) was used for comparison analysis, and data were presented using frequency and percentage. A comparison between continuous data for two independent groups was conducted using the Mann–Whitney *U*-test or independent-samples *T*-test. Cox proportional models were analyzed for determining the predictors of hospitalization for CV events. The findings were reported as a hazard ratio (HR) [95% confidence interval (CI)]. Statistically significant predictors in the univariate analysis were selected for multivariate analysis. Kaplan–Meier analysis with a log-rank test was performed to determine the effect of ACM related to the cumulative risk of hospitalization for CV events. The restricted mean survival time was the parameter used to estimate the expected value of time for patients to be free from CV events.

## Results

### Clinical characteristics of the study participants

A comparison of demographic and clinical variables between MetS patients with and without LVH is presented in Table 1. Overall, LVH accounted for 25.6% of patients hospitalized with MetS. The rate of prevalence of ACM in hospitalized patients who experienced MetS was 52.9%. The rates of ACM in hospitalized patients who presented with a normal LV size and LVH were 45.4% and 74.8%, respectively, suggesting that ACM was also found in a large proportion of MetS patients without LVH. Those with LVH had an increased burden of CVD-related risk factors compared with their non-LVH counterparts. The median age (62.3 vs. 59.8, *P *< 0.001), body mass index (26.47 vs. 26.16, *P *< 0.001), SBP (149 vs. 140, *P *< 0.001), DBP (86 vs. 81, *P *< 0.001), and creatinine (71 vs. 65, *P *< 0.001) were higher in MetS patients with LVH than in MetS patients with a normal left ventricle (*P *< 0.001). Likewise, the proportion of smokers (35.0% vs. 28.1%, *P *< 0.001) and alcohol consumers (24.8% vs. 19.3%, *P *< 0.001) was higher in MetS patients with LVH than in those without LVH. In addition, the rates of HTN (82.9% vs. 72.0%, *P *< 0.001), DM (40.4% vs. 32.4%, *P *< 0.001), and dyslipidemia (83.5% vs. 81.8%, *P *= 0.017) were significantly higher in the LVH group than in the non-LVH group (*P* < 0.05).

**Table 1:** Baseline characteristics.

Variables	All (15,528)	With LVH (3,976)	Without LVH (11,552)	*P*-value
Age, years	64.4 (57.5, 71.8)	62.3 (53.6, 69.5)	59.8 (51.2, 66.5)	<0.001
Male patients, *n* (%)	9,052 (58.3)	2,688 (67.6)	6,365 (55.1)	<0.001
BMI, kg/m^2^	26.24 (25.39, 28.43)	26.47 (25.53, 28.43)	26.16 (25.36, 27.81)	<0.001
Smokers, *n* (%)	4,281 (29.9)	1,272 (35.0)	3,009 (28.1)	<0.001
Alcohol consumption, *n* (%)	2,862 (20.7)	867 (24.8)	1,995 (19.3)	<0.001
HTN, *n* (%)	11,611 (74.8)	3,295 (82.9)	8,312 (72.0)	<0.001
SBP, mmHg	140 (130, 156)	149 (133,165)	140 (129, 151)	<0.001
DBP, mmHg	83 (76, 91)	86 (78, 97)	81 (75, 90)	<0.001
DM, *n* (%)	5,353 (34.5)	1,605 (40.4)	3,748 (32.4)	<0.001
Dyslipidemia, *n* (%)	12,776 (82.3)	3,321 (83.5)	9,455 (81.8)	0.017
TC, mmol/L	4.87 (4.18, 5.62)	4.84 (4.14, 5.63)	4.88 (4.19, 5.62)	0.270
TG, mmol/L	1.76 (1.24, 2.42)	1.80 (1.29, 2.54)	1.75 (1.23, 2.38)	<0.001
HDL, mmol/L	1.14 (0.96, 1.36)	1.11 (0.94, 1.35)	1.14 (0.96, 1.37)	0.001
LDL, mmol/L	2.82 (2.35, 3.34)	2.79 (2.34, 3.32)	2.83 (2.35, 3.35)	0.355
Atrial myopathy, *n* (%)	8,222 (52.9)	2,976 (74.8)	5,246 (45.4)	<0.001
PTFV1 < −4,000 uV*ms, *n* (%)	3,735 (24.1)	1,324 (33.3)	2,411 (20.9)	<0.001
LA enlargement, *n* (%)	6,687 (43.1)	2,644 (66.5)	4,043 (35.0)	<0.001
Ptfv1, 4,000 uV*ms	−2,418 (−3,906, −1,015)	−2,961 (−4,640, −1,368)	−2,256 (−3,654, −936)	<0.001
LA diameter, mm	37 (35, 39)	38 (36, 40)	36 (34, 38)	<0.001
LVEF, %	60.59 ± 3.35	60.44 ± 3.54	60.64 ± 3.28	0.002
Lp(a), mg/L	124 (65, 237)	131 (67, 244)	123 (65, 233)	0.029
Creatinine, μmol/L	67 (56, 78)	71 (59, 84)	65 (55, 76)	<0.001
Medications, *n* (%)				
Antihypertension, *n* (%)	9,426 (60.7)	2,734 (68.8)	6,692 (57.9)	<0.001
ACEIs/ARBs	2,068 (13.3)	666 (16.8)	1,402 (12.1)	<0.001
β-blockers	6,117 (39.4)	1,788 (45.0)	4,329 (37.5)	<0.001
Calcium antagonists	6,205 (40.0)	2,108 (53.0)	4,097 (35.5)	<0.001
Diuretics	2,122 (13.7)	827 (20.8)	1,295 (11.2)	<0.001
Antidiabetic drugs, *n* (%)	4,707 (30.3)	1,413 (35.6)	3,294 (28.5)	<0.001
Lipid-lowering drugs, *n* (%)	9,892 (63.7)	2,726 (67.3)	7,216 (62.5)	<0.001

LVH, left ventricular hypertrophy; BMI, body mass index; HTN, hypertension; SBP, systolic blood pressure; DBP, diastolic blood pressure; DM, diabetes mellitus; TC, total cholesterol; TG, triglycerides; LDL-C, low-density lipoprotein cholesterol; HDL-C, high-density lipoprotein cholesterol; ACM, atrial myopathy; LA, left atrium; PTFV1, P-wave terminal force in V1; LVEF, left ventricular ejection fraction; Lp(a), Lipoprotein(a); CV, cardiovascular; ACEI, angiotensin-converting enzyme inhibitor; ARB, angiotensin II receptor blocker; CCB, calcium channel blocker.

As shown in Table 2, the incidence of admission for CV events is slightly higher in the LVH group (49.3%) than in patients with a normal left ventricle size (47.1%). Among the LVH group, hospital admission due to CV events was more common among the older patients (63.05 vs. 61.55, *P *< 0.001). Admitted patients with LVH also had an increased likelihood of having ACM (79.2% vs. 70.6%, *P *< 0.001). Moreover, the use of antihypertensive (57.0% vs. 80.2%, *P *< 0.001), antidiabetic (32.3% vs. 38.8%, *P *< 0.001), and lipid-lowering medications (58.9% vs. 75.7%, *P *< 0.001) was relatively less common in hospitalized patients with LVH. Similarly, the prevalence of HTN (78.1% vs., 87.5%, *P *< 0.001), DM (38.2% vs. 42.4%, *P *< 0.001), and dyslipidemia (80.4% vs. 86.5%, *P *< 0.001) was significantly lower among the hospitalized patients experiencing MetS with LVH.

**Table 2:** Baseline characteristics in patients with and without atrial myopathy grouped by those with and without LVH.

Variables	With LVH			Without LVH		
	No readmission	Readmission	*P*-value	No readmission	Readmission	*P*-value
	(2,019)	(1,957)		(6,041)	(5,511)	
Age, years	61.55 (52.60, 68.49)	63.05 (54.59, 70.52)	<0.001	58.70 (49.56, 65.89)	60.68 (52.87, 67.21)	<0.001
Male patients, *n* (%)	1,390 (68.8)	1,298 (66.3)	0.090	3,411 (56.5)	2,954 (53.6)	0.002
Smoker, *n* (%)	666 (37.1)	606 (32.9)	0.007	1,569 (28.8)	1,440 (27.5)	0.127
Alcohol consumption, *n* (%)	447 (26.1)	420 (23.6)	0.095	1,033 (19.7)	962 (19.0)	0.385
BMI, kg/m^2^	26.47 (25.52, 28.41)	26.47 (25.54, 28.46)	0.828	26.17 (25.37, 27.81)	26.16 (25.35, 27.81)	0.390
HTN, *n* (%)	1,767 (87.5)	1,528 (78.1)	<0.001	4,633 (76.7)	3,683 (66.8)	<0.001
DBP, mmHg	89 (80, 99)	89 (80, 99)	0.078	82 (79, 90)	84 (78, 92)	0.001
SBP, mmHg	150 (138,165)	150 (139, 168)	0.017	140 (130, 152)	140 (130, 154)	0.005
DM, *n* (%)	857 (42.4)	748 (38.2)	0.007	2,047 (33.9)	1,701 (30.9)	0.001
Dyslipidemia, *n* (%)	1,747 (86.5)	1,574 (80.4)	<0.001	5,136 (85.0)	4,319 (78.4)	<0.001
TC, mmol/L	4.81 (4.12, 5.64)	4.86 (4.17, 5.62)	0.493	4.87 (4.15, 5.59)	4.89 (4.21, 5.66)	0.087
TG, mmol/L	1.72 (1.23, 2.37)	1.85 (1.35, 2.67)	0.001	1.66 (1.19, 2.30)	1.83 (1.28, 2.46)	<0.001
HDLC, mmol/L	1.16 (0.98, 1.38)	1.07 (0.91, 1.29)	<0.001	1.19 (1.00, 1.40)	1.10 (0.93, 1.32)	<0.001
LDLC, mmol/L	2.76 (2.28, 3.29)	2.81 (2.38, 3.35)	0.146	2.80 (2.33, 3.32)	2.85 (2.38, 3.37)	0.003
ACM, *n* (%)	1,426 (70.6)	1,550 (79.2)	<0.001	2,497 (41.3)	2,749 (49.9)	<0.001
LA enlargement, *n* (%)	1,280 (63.4)	1,364 (69.7)	<0.001	1,984 (32.8)	2,059 (37.4)	<0.001
PTFV1 < −4,000 uV*ms, *n* (%)	730 (37.3)	1,324 (33.3)	0.001	1,114 (18.4)	1,297 (23.5)	<0.001
LA diameter, mm	38 (36,40)	38 (36, 41)	<0.001	36 (34, 38)	36 (34, 39)	<0.001
PTFV1, 4,000 uV*ms	−2,666 (−4,399,−1,014)	−3,182 (−4,891, −1,833)	<0.001	−2,006 (−3,408, −720)	−2,535 (−3,886, −1,248)	<0.001
LVEF, %	60.41 ± 3.55	60.48 ± 3.53	0.523	60.67 ± 3.27	60.61 ± 3.29	0.356
Lp(a), mg/L	127 (66,244)	134 (68, 246)	0.530	121 (64,233)	127 (65, 235)	0.428
Creatinine, μmol/L	71 (60,83)	70 (59, 85)	0.832	65 (55,76)	65 (54, 75)	0.009
Medication, *n* (%)						
Antihypertriton, *n* (%)	1,619 (80.2)	1,115 (57.0)	<0.001	4,185 (69.3)	2,507 (45.5)	<0.001
ACEI	396 (19.6)	270 (13.8)	<0.001	857 (14.2)	545 (9.9)	<0.001
β-blockers	1,129 (55.9)	659 (33.7)	<0.001	2,796 (46.3)	1,533 (27.8)	<0.001
CCB	1,233 (61.1)	875 (44.7)	<0.001	2,505 (41.5)	1,592 (28.9)	<0.001
Diuretics	453 (22.4)	374 (19.1)	0.010	743 (12.3%)	552 (10.0)	<0.001
Antidiabetic drugs	783 (38.8)	630 (32.2)	<0.001	1,824 (30.2)	1,470 (26.7)	<0.001
Lipid-lowering drugs	1,524 (75.5)	1,152 (58.9)	<0.001	4,297 (71.1)	2,919 (53.0)	<0.001

LVH, left ventricular hypertrophy; BMI, body mass index; HTN, hypertension; SBP, systolic blood pressure; DBP, diastolic blood pressure; DM, diabetes mellitus; TC, total cholesterol; TG, triglycerides; LDL-C, low-density lipoprotein cholesterol; HDL-C, high-density lipoprotein cholesterol; ACM, atrial myopathy; LA, left atrium; PTFV1, P-wave terminal force in V1; LVEF, left ventricular ejection fraction; Lp(a), Lipoprotein(a); CV, cardiovascular; ACEI, angiotensin-converting enzyme inhibitor; ARB, angiotensin II receptor blocker; CCB, calcium channel blocker.

Among patients who had MetS with LVH, CVD-related risk factors were more common among the admission group. For example, patients who were admitted were older (60.68 vs. 58.70, *P *< 0.001) and presented with ACM (49.9% vs. 41.3%, *P *< 0.001). Surprisingly, patients with admission for CV events were less likely to have HTN (76.7% vs. 66.8%, *P *< 0.001), DM (33.9% vs. 30.9%, *P *< 0.001), and dyslipidemia (85.0% vs. 78.4%, *P *< 0.001), which may be attributed to the greater use of antihypertensive (45.4% vs. 69.3%, *P *< 0.001), antidiabetic (26.7% vs. 30.2%, *P *< 0.001), and lipid-lowering (53.0% vs. 71.1%, *P *< 0.001) treatment in the admission group than in their counterparts.

### Atrial cardiomyopathy for the prediction of cardiovascular disease-related readmissions

After a follow-up period of 33.2 ± 20.6 months, 7,468 (48.1%) patients were readmitted for CV events. The total duration of follow-up accounted for 43,049 person-years. Table 3 shows the results from the Cox proportional hazards model, revealing that ACM (HR, 1.29; 95% CI, 1.142–1.458) was associated with an increased risk of admission for CV events in patients with MetS and LVH. Also, ACM was found to be independently associated with the incidence of readmission due to CVD-related events in patients with MetS but with a normal LV size (HR, 1.175; 95% CI, 1.105–1.250). Moreover, factors such as older age, HTN, dyslipidemia, increased creatine levels, and poor adherence to antihypertensive drugs were associated with an increased likelihood of hospital admission. Among these variables, HTN accounted for the highest risk of admission in those patients experiencing MetS with a normal left ventricle size (HR, 1.634; 95% CI, 1.499–1.778) and LVH (HR, 1.521; 95% CI, 1.29–1.784). In addition, non-compliance with lipid-lowering and antidiabetic drugs significantly increased the risk of hospital admission due to CV events among those with Mets but with a normal left ventricle size.

**Table 3:** Univariate and multivariate COX analysis predictors of admission for cardiovascular events.

Variables	Univariate analysis						Multivariate analysis					
	With LVH			Without LVH			With LVH			Without LVH		
	HR	95% CI	*P*-value	HR	95% CI	*P*-value	HR	95% CI	*P*-value	HR	95% CI	*P*-value
Age	1.009	1.005–1.013	<0.001	1.013	1.011–1.015	<0.001	1.009	1.005–1.014	<0.001	1.016	1.013–1.019	<0.001
Gender	0.959	0.873–1.054	0.384	0.974	0.924–1.027	0.332	1.010	1.005–1.014	0.873	1.006	0.933–1.086	0.871
Smoker	0.893	0.810–0.984	0.023	0.965	0.909–1.026	0.256	0.959	0.830–1.108	0.569	1.039	0.945–1.142	0.427
Alcohol	0.917	0.822–1.023	0.120	0.971	0.905–1.041	0.405	0.992	0.853–1.153	0.917	1.108	0.992–1.123	0.725
BMI	1.001	0.982–1.020	0.908	0.990	0.977–1.004	0.151	1.015	0.993–1.038	0.189	1.003	0.987–1.018	0.739
ACM	1.374	1.232–1.533	<0.001	1.283	1.217–1.352	<0.001	1.290	1.142–1.458	<0.001	1.175	1.105–1.250	<0.001
LVEF	0.996	0.984–1.009	0.566	0.994	0.986–1.003	0.176	1.007	0.992–1.021	0.355	0.993	0.984–1.002	0.150
HTN	0.789	0.709–0.878	<0.001	0.806	0.752–0.853	<0.001	1.521	1.297–1.784	<0.001	1.634	1.499–1.778	<0.001
DM	0.879	0.803–0.963	<0.001	0.872	0.824–0.924	<0.001	1.089	0.873–1.357	0.450	0.852	0.725–1.001	0.051
Dyslipidemia	0.850	0.760–0.950	0.004	0.797	0.748–0.850	<0.001	1.223	1.041–1.438	0.015	1.147	1.045–1.259	0.004
Creatine	1.001	1.000–1.001	0.001	1.002	1.001–1.003	<0.001	1.001	1.001–1.002	<0.001	1.002	1.001–1.003	0.003
Antihypertension	0.595	0.544–0.650	<0.001	0.578	0.548–0.609	<0.001	0.484	0.417–0.562	<0.001	0.453	0.416–0.493	<0.001
Lipid-lowering	0.737	0.674–0.807	<0.001	0.687	0.651–0.724	<0.001	0.881	0.753–1.032	0.113	0.774	0.712–0.841	<0.001
Antidiabetics	0.824	0.750–0.906	<0.001	0.856	0.807–0.909	<0.001	0.889	0.713–1.135	0.371	1.242	1.049–1.470	0.012

LVH, left ventricular hypertrophy; BMI, body mass index; LVEF, left ventricular ejection fraction; ACM, atrial myopathy; LVH, left ventricular hypertrophy; HTN, hypertension; DM, diabetes mellitus; HR, hazard ratio; CI, confidence interval.

**Figure 2:**
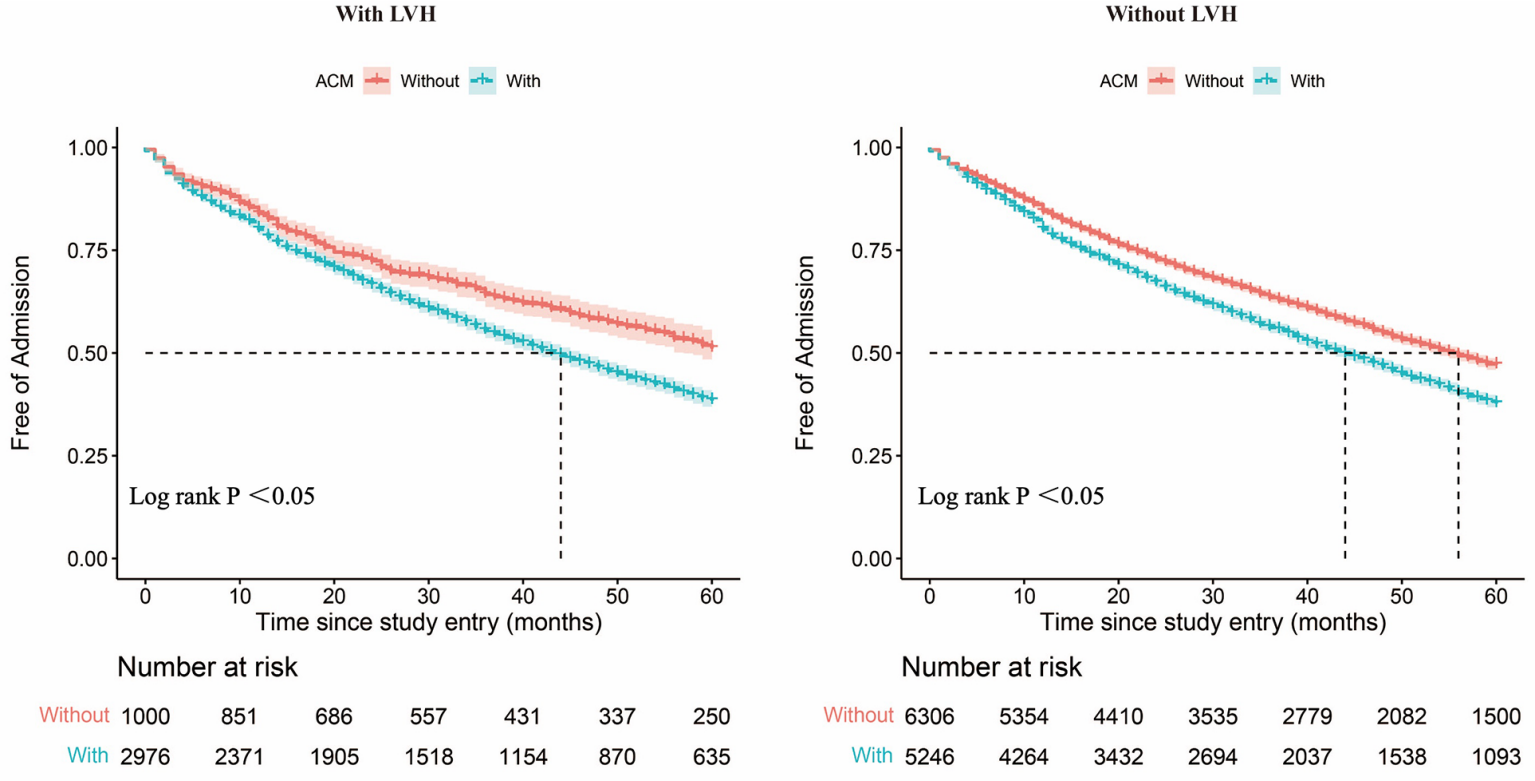
Admission-free survival curves. Admission-free survival curves for patients with and without the ACM group among patients with LVH. Admission-free survival curves for patients with and without the ACM group among patients without LVH. ACM, atrial myopathy; LVH, left ventricular hypertrophy.

**Figure 3:**
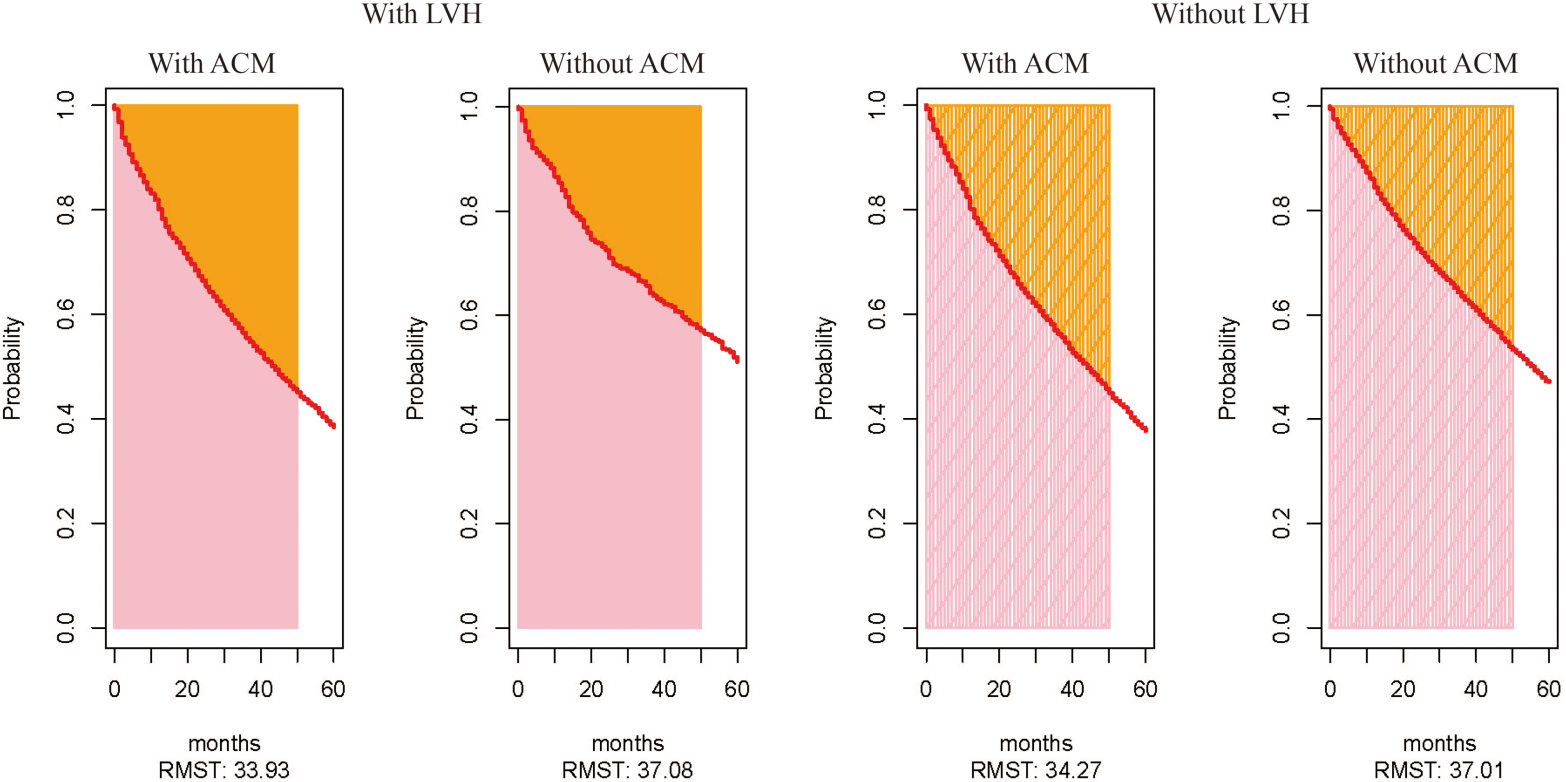
Expected values of time for patients to be free from CV events, calculated by using the parameter of restricted mean survival time. The expected value of time to be free from CV events for patients with and without ACM among those with LVH. The expected value of time to be free from CV events for patients with and without ACM among those without LVH.

Figures 2 shows the Kaplan–Meier curves for freedom for hospital admission in MetS patients with and without ACM, respectively. This result indicates that these individuals with ACM were more often rehospitalized for CVD compared with those without ACM. Also, the ACM group had a shorter free duration from hospitalization due to CVD events. Over the 5-year follow-up period, patients with MetS and LVH were expected to be free from hospital admission for 33.93 months if they suffered from ACM (95% CI, 33.26–34.60) and for 37.08 months (95% CI, 35.96–38.20) if they did not suffer from ACM (Figures 3). Also, patients with MetS but no evidence of LVH were expected to be free from hospital admission for 34.27 months (95% CI, 33.77–34.77) if they presented with ACM and for 37.01 months (95% CI, 36.57–37.45) if they were free from ACM.

## Discussion

Our study demonstrates that the prevalence of ACM is high in individuals with MetS. Notably, ACM was also significant in MetS patients without LVH (45.4%), which implied that ACM may be a marker of early myocardial remodeling in patients with MetS. In particular, we found that patients with ACM and MetS had an increased risk of hospitalization for CV events.

Historically, AF has been observed under prolonged hemodynamic stress, including HTN and valvular heart disease (29). Conversely, a recent study proposed a different theory and stated that metabolic diseases such as obesity, DM, and systemic inflammatory disorders associated with adipose tissue may be the most prominent antecedents of AF. In addition, recent studies have indicated that ACM may exist in the absence of AF and may facilitate the development of AF (30). In addition, ACM may be an underlying mechanism in the development of systemic thromboembolism (31). The combination of these contributing factors may explain the high prevalence of ACM in patients with MetS. Interestingly, 45.4% of the ACM patients with MetS were free of LVH, suggesting that ACM can be a marker of early myocardial remodeling in patients with MetS.

ACM is exclusively defined in terms of PTFV1 >4,000 uV·ms and the presence of severe LAE. It has been well established that abnormal PTFV1 is associated with LA abnormalities on the ECG (32). In the past, the diagnosis of ACM depended on the presence of structural or functional abnormalities of the LA on cardiac imaging (33). Although increased PTFV1 is thought to be a sign of LAE, it is more reliably a sign of delayed interarterial conduction (34). In the present study, some patients had an increase in PTFV1 without LAE, whereas some patients had an enlarged LA without an increase in PTFV1. Since both increased PTFV1 and LA diameter (LAD) have been correlated with elevated LA pressure, systemic HTN, ischemic heart disease, and prolonged interatrial conduction (31, 35), the interaction between PTFV1 and LAD needs to be elucidated.

In the past, a large Asianpopulation–based cohort reported a positive correlation between the components of MetS diagnostic criteria and the risk of AF (36). The association between the cumulative number of total MetS components and the risk of AF may suggest that upgrading efforts to identify and correct metabolic derangements even before the development of MetS could be of crucial importance to preventing ACM and related CVDs. However, in our study, we found that almost 26% of hospitalized patients with HTN and MetS did not undergo any antihypertensive therapy. Moreover, over 34% of individuals with MetS did not meet the recommended target SBP of less than 140 mmHg and target DBP pressure of less than 90 mmHg. In addition, the optimized management targeting DM and dyslipidemia was also found to be limited. Low awareness of the importance of lipid control strategies among non-cardiac departments may be a contributing factor. This finding highlights that there is still a need for optimizing blood pressure and lipid control in the inpatient management of hospitalized populations who are at risk for ACM.

The use of the early cardiac remodeling technique may be a reasonable proposition in patients with ACM. However, the association between increased instances of early cardiac remodeling and CV events requires further elucidation. Although it is beyond the scope of the present study to investigate the underlying biological mechanisms, it can be hypothesized that chronic inflammation may represent a triggering factor in the development of MetS, and recently, ACM in ischemic stroke has been demonstrated to correlate with the degree of chronic inflammation (37), which represents a possible pathogenic factor. It is well known that both obesity and diabetes promote a state of systemic inflammation that can lead to the expansion of epicardial adipose tissue, which becomes a source of proinflammatory secretory products that cause structural and functional abnormalities in the underlying myocardium (38). The expansion of epicardial fat in the LA, resulting in electroanatomic remodeling, could lead to ACM (39), which further predisposes to blood stasis, spontaneous thrombus formation, and stroke (40). Additionally, altered autonomic nervous system activity may be involved in the development of both MetS and ACM. It has been proven that there is a close link between the autonomic nervous system (41) and MetS. The alterations in the autonomic nervous system also play an important role in atrial cardiopathy (42). Last but not least, other forms of adipose tissue inflammation and insulin resistance in Mets are accompanied by an increased risk of atrial remodeling (43). All in all, the relevant roles of many factors contribute to the development of both ACM and MetS.

## Limitations

There are several limitations to this study. Firstly, this retrospective study was carried out in a single center, and therefore, collection and registration bias may be present. Secondly, no implantable loop monitoring was performed in MetS patients, which may lead to an underestimation of AF occurrence. Lastly, only recorded indicators and variables are included; unregistered significant variables may have been omitted. Despite these limitations, our study was the first to our knowledge to investigate the prevalence of ACM in hospitalized patients with MetS and demonstrated that ACM is a common condition that could predict hospital admissions for CV events.

## Conclusion

To conclude, ACM may be a marker of early myocardial remodeling in patients with MetS and predicts CV-related hospital admissions. Therefore, there is a need for further optimization in the management of ACM in a hospital setting.

## Data Availability

The raw data supporting the conclusions of this article will be made available by the authors without undue reservation.

## References

[1] Kornej J Borschel CS Benjamin EJ Schnabel RB. Epidemiology of atrial fibrillation in the 21st century: novel methods and new insights. Circ Res. (2020) 127(1):4–20. 10.1161/CIRCRESAHA.120.316340 32716709 PMC7577553

[2] Grundy SM Cleeman JI Daniels SR Donato KA Eckel RH Franklin BA Diagnosis and management of the metabolic syndrome: an American Heart Association/National Heart, Lung, and Blood Institute Scientific Statement. Circulation. (2005) 112(17):2735–52. 10.1161/CIRCULATIONAHA.105.169404 16157765

[3] Expert Panel on Detection, Evaluation, and Treatment of High Blood Cholesterol in Adults. Executive summary of the third report of the National Cholesterol Education Program (NCEP) expert panel on detection, evaluation, and treatment of high blood cholesterol in adults (adult treatment panel III). JAMA. (2001) 285(19):2486–97. 10.1001/jama.285.19.2486 11368702

[4] Hajhosseiny R Matthews GK Lip GY. Metabolic syndrome, atrial fibrillation, and stroke: tackling an emerging epidemic. Heart Rhythm. (2015) 12(11):2332–43. 10.1016/j.hrthm.2015.06.038 26142297

[5] Devereux RB Alderman MH. Role of preclinical cardiovascular disease in the evolution from risk factor exposure to development of morbid events. Circulation. (1993) 88(4 Pt 1):1444–55. 10.1161/01.cir.88.4.1444 8403291

[6] Kamel H Okin PM Longstreth WT Elkind MSV Soliman EZ. Atrial cardiopathy: a broadened concept of left atrial thromboembolism beyond atrial fibrillation. Future Cardiol. (2015) 11(3):323–31. 10.2217/fca.15.22 26021638 PMC4868349

[7] Vaziri SM Larson MG Benjamin EJ Levy D. Echocardiographic predictors of nonrheumatic atrial fibrillation. The Framingham Heart Study. Circulation. (1994) 89(2):724–30. 10.1161/01.cir.89.2.724 8313561

[8] Frustaci A Chimenti C Bellocci F Morgante E Russo MA Maseri A. Histological substrate of atrial biopsies in patients with lone atrial fibrillation. Circulation. (1997) 96(4):1180–4. 10.1161/01.cir.96.4.1180 9286947

[9] Cai H Li Z Goette A Mera F Honeycutt C Feterik K Downregulation of endocardial nitric oxide synthase expression and nitric oxide production in atrial fibrillation: potential mechanisms for atrial thrombosis and stroke. Circulation. (2002) 106(22):2854–8. 10.1161/01.cir.0000039327.11661.16 12451014

[10] Acampa M Lazzerini PE Guideri F Tassi R Cartocci A Martini G. P wave dispersion and silent atrial fibrillation in cryptogenic stroke: the pathogenic role of inflammation. Cardiovasc Hematol Disord Drug Targets. (2019) 19(3):249–52. 10.2174/1871529X19666190410145501 30968778

[11] Kamel H Soliman EZ Heckbert SR Kronmal RA Longstreth WT Nazarian S P-wave morphology and the risk of incident ischemic stroke in the multi-ethnic study of atherosclerosis. Stroke. (2014) 45(9):2786–8. 10.1161/STROKEAHA.114.006364 25052322 PMC4146624

[12] Kamel H Elkind MSV Bhave PD Navi BB Okin PM Iadecola C Paroxysmal supraventricular tachycardia and the risk of ischemic stroke. Stroke. (2013) 44(6):1550–4. 10.1161/STROKEAHA.113.001118 23632982 PMC3950597

[13] Binici Z Intzilakis T Nielsen OW Køber L Sajadieh A. Excessive supraventricular ectopic activity and increased risk of atrial fibrillation and stroke. Circulation. (2010) 121(17):1904–11. 10.1161/CIRCULATIONAHA.109.874982 20404258

[14] Sebasigari D Merkler A Guo Y Gialdini G Kummer B Hemendinger M Biomarkers of atrial cardiopathy and atrial fibrillation detection on mobile outpatient continuous telemetry after embolic stroke of undetermined source. J Stroke Cerebrovasc Dis. (2017) 26(6):1249–53. 10.1016/j.jstrokecerebrovasdis.2017.01.016 28237125

[15] Benjamin EJ D'Agostino RB Belanger AJ Wolf PA Levy D. Left atrial size and the risk of stroke and death. The Framingham Heart Study. Circulation. (1995) 92(4):835–41. 10.1161/01.CIR.92.4.835 7641364

[16] Folsom AR Nambi V Bell EJ Oluleye OW Gottesman RF Lutsey PL Troponin T, N-terminal pro-B-type natriuretic peptide, and incidence of stroke: the atherosclerosis risk in communities study. Stroke. (2013) 44(4):961–7. 10.1161/STROKEAHA.111.000173 23471272 PMC3614093

[17] Hoit BD. Left atrial size and function: role in prognosis. J Am Coll Cardiol. (2014) 63(6):493–505. 10.1016/j.jacc.2013.10.055 24291276

[18] Liu F Hidru TH Gao R Lin Y Liu Y Fang F Cancer patients with potential eligibility for vascular endothelial growth factor antagonists use have an increased risk for cardiovascular diseases comorbidities. J Hypertens. (2020) 38(3):426–33. 10.1097/HJH.0000000000002277 31584518 PMC7012358

[19] Third report of the National Cholesterol Education Program (NCEP) expert panel on detection, evaluation, and treatment of high blood cholesterol in adults (adult treatment panel III) final report. Circulation. (2002) 106(25):3143–421. 10.1161/circ.106.25.3143 12485966

[20] Mitchell C Rahko PS Blauwet LA Canaday B Finstuen JA Foster MC Guidelines for performing a comprehensive transthoracic echocardiographic examination in adults: recommendations from the American Society of Echocardiography. J Am Soc Echocardiogr. (2019) 32(1):1–64. 10.1016/j.echo.2018.06.004 30282592

[21] Hidru TH Tang Y Liu F Hui S Gao R Li D Does serum uric acid status influence the association between left atrium diameter and atrial fibrillation in hypertension patients? Front Cardiovasc Med. (2020) 7:594788. 10.3389/fcvm.2020.594788 33330657 PMC7732653

[22] Devereux RB Alonso DR Lutas EM Gottlieb GJ Campo E Sachs I Echocardiographic assessment of left ventricular hypertrophy: comparison to necropsy findings. Am J Cardiol. (1986) 57(6):450–8. 10.1016/0002-9149(86)90771-x 2936235

[23] Lang RM Bierig M Devereux RB Flachskampf FA Foster E Pellikka PA Recommendations for chamber quantification: a report from the American Society of Echocardiography's Guidelines and Standards Committee and the Chamber Quantification Writing Group, developed in conjunction with the European Association of Echocardiography, a branch of the European Society of Cardiology. J Am Soc Echocardiogr. (2005) 18(12):1440–63. 10.1016/j.echo.2005.10.005 16376782

[24] Elkind MSV. Atrial cardiopathy and stroke prevention. Current cardiology reports. (2018) 20(11):103. 10.1007/s11886-018-1053-0 30209635

[25] Jin L Weisse AB Hernandez F Jordan T. Significance of electrocardiographic isolated abnormal terminal P-wave force (left atrial abnormality). An echocardiographic and clinical correlation. Arch Intern Med. (1988) 148(7):1545–9. 10.1001/archinte.1988.00380070053014 2968074

[26] Williams B Mancia G Spiering W Agabiti Rosei E Azizi M Burnier M 2018 ESC/ESH guidelines for the management of arterial hypertension. Eur Heart J. (2018) 39(33):3021–104. 10.1093/eurheartj/ehy339 30165516

[27] Joint committee for guideline revision. 2016 Chinese guidelines for the management of dyslipidemia in adults. J Geriatr Cardiol. (2018) 15(1):1–29. 10.11909/j.issn.1671-5411.2018.01.011 29434622 PMC5803534

[28] Pearson TA Palaniappan LP Artinian NT Carnethon MR Criqui MH Daniels SR American Heart Association Guide for Improving Cardiovascular Health at the Community Level, 2013 update: a scientific statement for public health practitioners, healthcare providers, and health policy makers. Circulation. (2013) 127(16):1730–53. 10.1161/CIR.0b013e31828f8a94 23519758

[29] Lévy S. Factors predisposing to the development of atrial fibrillation. Pacing Clin Electrophysiol. (1997) 20(10 Pt 2):2670–4. 10.1111/j.1540-8159.1997.tb06115.x 9358513

[30] Kottkamp H. Fibrotic atrial cardiomyopathy: a specific disease/syndrome supplying substrates for atrial fibrillation, atrial tachycardia, sinus node disease, AV node disease, and thromboembolic complications. J Cardiovasc Electrophysiol. (2012) 23(7):797–9. 10.1111/j.1540-8167.2012.02341.x 22554187

[31] Russo C Jin Z Liu R Iwata S Tugcu A Yoshita M LA volumes and reservoir function are associated with subclinical cerebrovascular disease: the CABL (Cardiovascular Abnormalities and Brain Lesions) study. JACC Cardiovasc Imaging. (2013) 6(3):313–23. 10.1016/j.jcmg.2012.10.019 23473112 PMC3600634

[32] Morris JJ Jr. Estes EH Jr. Whalen RE Thompson HK Jr. McIntosh HD. P-wave analysis in valvular heart disease. Circulation. (1964) 29:242–52. 10.1161/01.cir.29.2.242 14119389

[33] Hirsh BJ Copeland-Halperin RS Halperin JL. Fibrotic atrial cardiomyopathy, atrial fibrillation, and thromboembolism: mechanistic links and clinical inferences. J Am Coll Cardiol. (2015) 65(20):2239–51. 10.1016/j.jacc.2015.03.557 25998669

[34] Josephson ME Kastor JA Morganroth J. Electrocardiographic left atrial enlargement. Electrophysiologic, echocardiographic and hemodynamic correlates. Am J Cardiol. (1977) 39(7):967–71. 10.1016/S0002-9149(77)80209-9 141202

[35] Chen LY Soliman EZ. P wave indices-advancing our understanding of atrial fibrillation-related cardiovascular outcomes. Front Cardiovasc Med. (2019) 6:53. 10.3389/fcvm.2019.00053 PMC6509260 31131284

[36] Ahn HJ Han KD Choi EK Jung JH Kwon S Lee SR Cumulative burden of metabolic syndrome and its components on the risk of atrial fibrillation: a nationwide population-based study. Cardiovasc Diabetol. (2021) 20(1):20. 10.1186/s12933-021-01215-8 33468142 PMC7816376

[37] Acampa M Lazzerini PE Guideri F Tassi R Lo Monaco A Martini G. Inflammation and atrial electrical remodelling in patients with embolic strokes of undetermined source. Heart Lung Circ. (2019) 28(6):917–22. 10.1016/j.hlc.2018.04.294 29887417

[38] Packer M. Epicardial adipose tissue may mediate deleterious effects of obesity and inflammation on the myocardium. J Am Coll Cardiol. (2018) 71(20):2360–72. 10.1016/j.jacc.2018.03.509 29773163

[39] Mahajan R Nelson A Pathak RK Middeldorp ME Wong CX Twomey DJ Electroanatomical remodeling of the atria in obesity: impact of adjacent epicardial fat. JACC Clin Electrophysiol. (2018) 4(12):1529–40. 10.1016/j.jacep.2018.08.014 30573116

[40] Calenda BW Fuster V Halperin JL Granger CB. Stroke risk assessment in atrial fibrillation: risk factors and markers of atrial myopathy. Nat Rev Cardiol. (2016) 13(9):549–59. 10.1038/nrcardio.2016.106 27383079

[41] Yu TY Lee MK. Autonomic dysfunction, diabetes and metabolic syndrome. J Diabetes Investig. (2021) 12(12):2108–11. 10.1111/jdi.13691 34622579 PMC8668070

[42] Acampa M Lazzerini PE Martini G. Atrial cardiopathy and sympatho-vagal imbalance in cryptogenic stroke: pathogenic mechanisms and effects on electrocardiographic markers. Front Neurol. (2018) 9:469. 10.3389/fneur.2018.00469 29971041 PMC6018106

[43] Kwon CH Kim H Kim SH Kim BS Kim H-J Kim D-K The impact of metabolic syndrome on the incidence of atrial fibrillation: a nationwide longitudinal cohort study in South Korea. J Clin Med. (2019) 8(8):1095. 10.3390/jcm8081095 PMC6723247 31344944

